# Lung Microbiota Changes Associated with Chronic *Pseudomonas aeruginosa* Lung Infection and the Impact of Intravenous Colistimethate Sodium

**DOI:** 10.1371/journal.pone.0142097

**Published:** 2015-11-06

**Authors:** David Collie, Laura Glendinning, John Govan, Steven Wright, Elisabeth Thornton, Peter Tennant, Catherine Doherty, Gerry McLachlan

**Affiliations:** 1 The Roslin Institute and Royal (Dick) School of Veterinary Studies, University of Edinburgh, Edinburgh, United Kingdom; 2 University of Edinburgh, Medical School, Edinburgh, Scotland, United Kingdom; Purdue University, UNITED STATES

## Abstract

**Background:**

Exacerbations associated with chronic lung infection with *Pseudomonas aeruginosa* are a major contributor to morbidity, mortality and premature death in cystic fibrosis. Such exacerbations are treated with antibiotics, which generally lead to an improvement in lung function and reduced sputum *P*. *aeruginosa* density. This potentially suggests a role for the latter in the pathogenesis of exacerbations. However, other data suggesting that changes in *P*. *aeruginosa* sputum culture status may not reliably predict an improvement in clinical status, and data indicating no significant changes in either total bacterial counts or in *P*. *aeruginosa* numbers in sputum samples collected prior to pulmonary exacerbation sheds doubt on this assumption. We used our recently developed lung segmental model of chronic *Pseudomonas* infection in sheep to investigate the lung microbiota changes associated with chronic *P*. *aeruginosa* lung infection and the impact of systemic therapy with colistimethate sodium (CMS).

**Methodology/Principal Findings:**

We collected protected specimen brush (PSB) samples from sheep (n = 8) both prior to and 14 days after establishment of chronic local lung infection with *P aeruginosa*. Samples were taken from both directly infected lung segments (direct) and segments spatially remote to such sites (remote). Four sheep were treated with daily intravenous injections of CMS between days 7 and 14, and four were treated with a placebo. Necropsy examination at d14 confirmed the presence of chronic local lung infection and lung pathology in every direct lung segment.

The predominant orders in lung microbiota communities before infection were Bacillales, Actinomycetales and Clostridiales. While lung microbiota samples were more likely to share similarities with other samples derived from the same lung, considerable within- and between-animal heterogeneity could be appreciated.

Pseudomonadales joined the aforementioned list of predominant orders in lung microbiota communities after infection. Whilst treatment with CMS appeared to have little impact on microbial community composition after infection, or the change undergone by communities in reaching that state, when Gram negative organisms (excluding Pseudomonadales) were considered together as a group there was a significant decrease in their relative proportion that was only observed in the sheep treated with CMS. With only one exception the reduction was seen in both direct and remote lung segments. This reduction, coupled with generally increasing or stable levels of Pseudomonadales, meant that the proportion of the latter relative to total Gram negative bacteria increased in all bar one direct and one remote lung segment.

**Conclusions/Significance:**

The proportional increase in Pseudomonadales relative to other Gram negative bacteria in the lungs of sheep treated with systemic CMS highlights the potential for such therapies to inadvertently select or create a niche for bacteria seeding from a persistent source of chronic infection.

## Introduction

*Pseudomonas aeruginosa* is considered to be the most important pathogen in cystic fibrosis (CF), with up to 60% of adult patients infected (UK CF Registry Annual Data Report 2014 [1]), and is also frequently isolated from patients with bronchiectasis [2]. In CF, early infections with *P*. *aeruginosa* can be transient, and can clear spontaneously, but colonization with *P*. *aeruginosa* usually occurs by the time patients reach their teenage years. In the later stages of infection, there is an adaptive shift from free-swimming planktonic *P*. *aeruginosa* to a sessile biofilm mode involving mucoid alginate-producing variants of the original colonising strain [3]. This important and characteristic shift is associated with more frequent and more severe pulmonary exacerbations (PEs) that result in progressive decrements in lung function [4].

*P*. *aeruginosa* also dominates chronic infections in a proportion of patients with bronchiectasis [5] and in chronic obstructive pulmonary disease (COPD) [6] where there is an increasing association with acute exacerbations.

The factors linking chronic *P*. *aeruginosa* lung infection to PEs are currently unknown. Certainly studies indicating that there is a reduction of sputum *P*. *aeruginosa* density during antibiotic treatment for PE in CF patients—a change that correlates with an improvement in lung function [7], tend to support a primary role for *P aeruginosa*. However, other data suggesting that changes in *P*. *aeruginosa* sputum culture status may not reliably predict an improvement in clinical status [8], and data indicating no significant changes in either total bacterial counts or in *P*. *aeruginosa* numbers in sputum samples collected prior to pulmonary exacerbation [9] sheds doubt on the specific role of *P*. *aeruginosa* in PE. Such uncertainty has been added to by recent 16S ribosomal DNA sequencing data. In a recent study of fifteen CF patients followed through 21 pulmonary exacerbations, sputum *P*. *aeruginosa* numbers did not increase immediately prior to a PE in CF adults [10]. These findings bear comparison with those of Carmody et al (2013) who found that during PE in CF patients bacterial community diversity and bacterial density in sputum samples did not change between baseline and exacerbation [11], and Price et al (2013) who similarly found that total and relative abundance of genera at the population level were remarkably stable for individual patients regardless of clinical status [12]. These studies indicate that there are no generalizable ecological ‘signatures’ of PE in this type of clinical sample.

Daniels et al (2013)[13] investigated the relative impact of antibiotics, used predominantly to target *P*. *aeruginosa* during acute exacerbations, on other non-pseudomonal species. The relative abundance of viable *P*. *aeruginosa* and non-pseudomonal species was determined in sputa from adult CF subjects in the days preceding an exacerbation, and during and after antibiotic therapy, by T-RFLP profiling. Overall, an increase in the relative abundance of *P*. *aeruginosa* was observed, with a decrease in the total number of species detected. They raised the possibility that, aside from the direct effect of systemic antimicrobials on *P*. *aeruginosa*, there is coincident impact of antimicrobials on the remaining community members such that unspecified changes to *P*. *aeruginosa* gene expression may occur as a result of changes in interspecies communication. The potential exists for such changes in gene expression to impact on virulence and/or persistence [14].

Much of our current perceptions relating to the pathogenesis of PEs are driven by such studies relying on sputum to monitor inflammatory cells, bacterial densities, volatiles, mucin and protein content of the airways [15]. However, sputum characteristics can be highly variable between subjects and even within apparently stable subjects over time–reflecting, at least in part, heterogeneity of pathology across different lung regions and/or the relative contribution of different regions to the final sputum volume. Therefore sputum at best represents an averaging process, and at worst provides a highly skewed view of lung physiology and pathophysiology. These limitations may critically undermine our ability to u0nderstand the way in which heterogeneous disease processes, and associated microbiota, trigger fulminant whole-organ PEs.

Large animal models provide the means to dissect the pathophysiology of lung disease at a local level. Motivated by the current dearth of information surrounding the pathogenesis of PEs and speculation over the role of respiratory microbiota in this regard we recently developed a novel ovine model of chronic local lung infection with *P*. *aeruginosa* [16]. Our objectives in this research were to develop an understanding of the local pulmonary and microbiota response to chronic local lung infection with *P*. *aeruginosa* and to characterise the way in which systemic antibiotic therapy impacts on this response. We selected colistimethate sodium (CMS) as our antibiotic of choice. CMS undergoes hydrolysis in aqueous solutions to form a complex mixture of derivatives, including colistin [17]. Colistin is a polymyxin antibiotic with activity against Gram-negative organisms including *P*. *aeruginosa*. Whilst its use declined in the 1970s over concerns about toxicity it has recently experienced a resurgence as a consequence of the rise in resistance of *P*. *aeruginosa* and *Acinetobacter spp* to extended-spectrum cephalosporins, aminoglycosides, and fluoroquinolones. It is therefore a relevant choice in this context.

## Methods

### Ethics Statement

All experimental protocols were reviewed and approved by the local Roslin Institute ethical review process (The Roslin Institute Animal Welfare and Ethics Committee) and were subject to the provisions of the Animals (Scientific Procedures) Act 1986. During the course of the experimental protocols the animals were assessed on a daily basis for any clinical signs of adverse effect including dullness, depression, inappetence, coughing and/or dyspnoea.

### Animals

Eight Suffolk cross sheep (4F & 4MN; Bodyweight 40.5 [38–48] Kg (Median[Range])) were used in this study. These sheep were commercially sourced and housed in groups on straw bedding under standard management conditions appropriate to a research setting. Sheep were randomly assigned to one of two treatment groups (Placebo (n = 4) and colistimethate sodium (CMS)(n = 4)).

### Experimental Design

**Fig 1:**
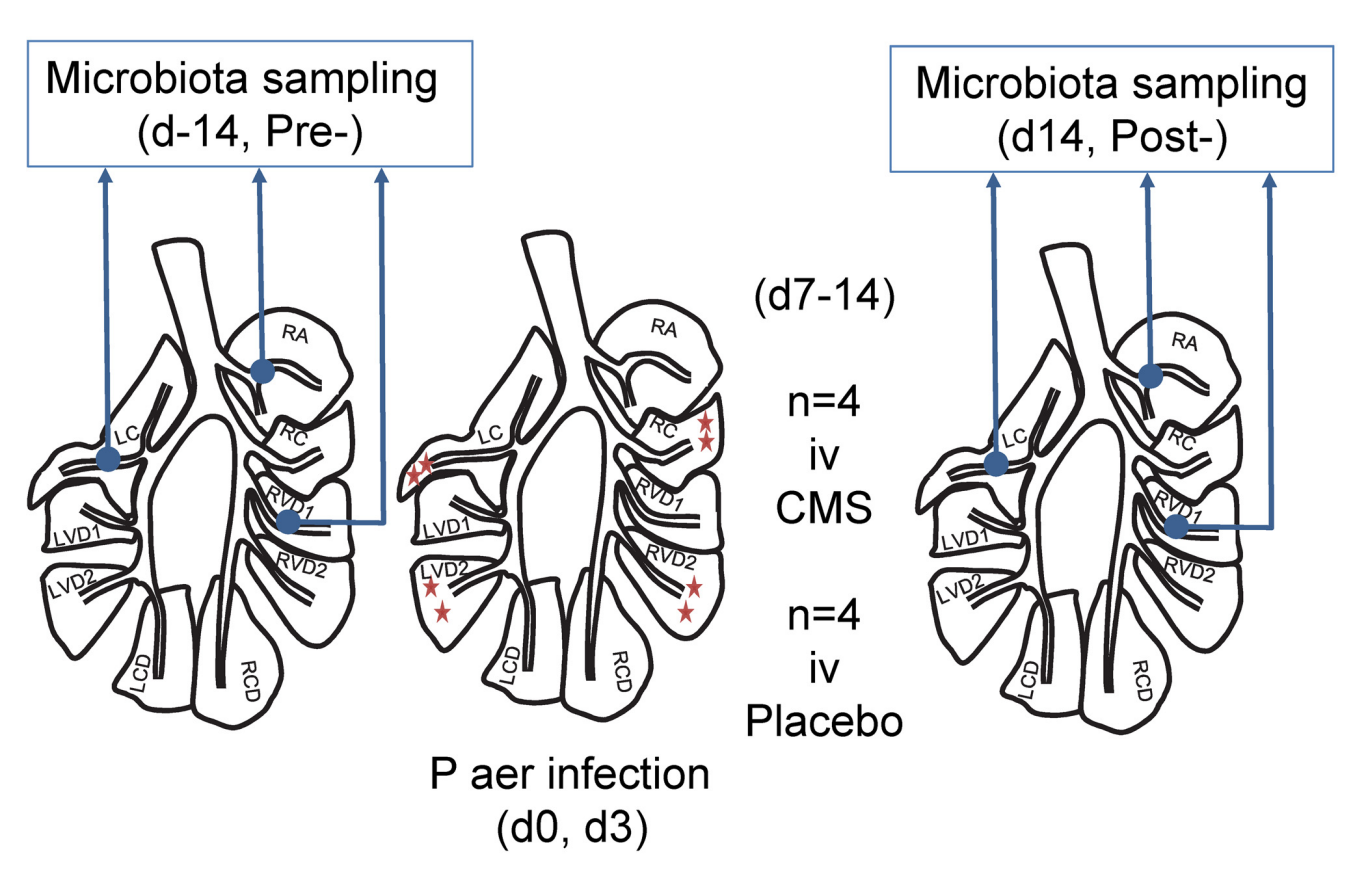
Microbiota sampling protocol. During a baseline examination lung health was confirmed in the form of direct visualisation of the airway tree and later cytological analysis of bronchoalveolar lavage fluid (BALF). Protected specimen brush (PSB) samples were collected from the segmental bronchus serving the right apical (RA) lobe, the first ventral diaphragmatic (RVD1) segment of the right caudal diaphragmatic lobe, and the left cardiac (LC) segment of the apicocardiac lobe (Pre-samples). BALF was subsequently collected from RA. At least two weeks later *P*. *aeruginosa* agar beads were instilled into the right cardiac (RC) lobe, the second ventral diaphragmatic (RVD2) segment of the right caudal diaphragmatic lobe, the left cardiac (LC) segment of the apicocardiac lobe, and the second ventral diaphragmatic (LVD2) segment of the left caudal diaphragmatic lobe. Three days later these instillations were repeated. Four days after the second instillation sheep were randomly assigned to daily intravenous injections of either CMS or placebo. Eleven days after the second instillation–after one week of daily injections—PSB specimens were obtained from the previously sampled lobes (RA, RVD1 and LC) (Post-samples). After the sheep was killed and the lungs removed for further analysis, BALF was derived from each segment under study (RA, RC, RVD1, RVD2, LC and LVD2) prior to further dissection, sampling and recording.

A baseline examination was conducted in which each sheep was subject to clinical examination and a blood sample taken from the jugular vein for routine haematological analysis. Thereafter each sheep was anaesthetised to facilitate bronchoscopic examination and sample collection according to standard protocol [16]. During this examination lung health was confirmed in the form of direct visualisation of the airway tree and later cytological analysis of bronchoalveolar lavage fluid (BALF). Protected specimen brush (PSB) samples were collected from the segmental bronchus serving the right apical (RA) lobe, the first ventral diaphragmatic (RVD1) segment of the right caudal diaphragmatic lobe, and the left cardiac (LC) segment of the apicocardiac lobe (Fig 1). BALF was subsequently collected from RA. After a recovery period of not less than two weeks (23 [15–34] days), the sheep were re-anaesthetised and Pseudomonas in agar beads (2.5x10^9^ cfu in 2.5ml) instilled into the right cardiac (RC) lobe, the second ventral diaphragmatic (RVD2) segment of the right caudal diaphragmatic lobe, the left cardiac (LC) segment of the apicocardiac lobe, and the second ventral diaphragmatic (LVD2) segment of the left caudal diaphragmatic lobe. The method of instillation followed that previously described [16]. Three days later the sheep were blood sampled, anaesthetised again, and these instillations repeated. Four days after the second instillation the sheep assigned to the CMS group commenced daily treatment with intravenously administered systemic antibiotic (an intravenous dose of 50,000 international units (IU) kg-1 of colistimethate sodium (Colomycin®, Forest Laboratories UK Ltd, Dartford, Kent) every 24 h), with the placebo group commencing daily injections of saline. Eleven days after the second instillation–after one week of daily injections—blood samples were acquired before the sheep were anaesthetised and PSB specimens obtained from the previously sampled lobes (RA, RVD1 and LC). The sheep were then euthanized by intravenous injection of baribiturate, and the heart and lungs carefully removed from the carcase following standard necropsy protocols before the heart was dissected away and the lungs presented for further sampling and analysis. BALF was derived from each segment under study (RA, RC, RVD1, RVD2, LC and LVD2) prior to further dissection, sampling and recording using previously described methodology [16]. This experimental protocol and sampling scheme therefore allowed us to evaluate the effects of local lung infection with P aer., both within the direct segments (RC, LC, RVD2 and LVD2), within non-infected segments remote to the sites of direct infection (RA and RVD1), and systemically.

### Anaesthesia

Food was withheld for 12 hours prior to anaesthesia. General anaesthesia was induced with intravenously injected propofol (6–8mg/kg)(Fresenius propofol, 1%, Fresenius Kabi Ltd) and anaesthesia maintained using positive pressure ventilation (Model 708; Harvard Apparatus, Millis, MA) with a 2:1 mixture of oxygen and nitrous oxide, and 1–3% isoflurane. Tidal volume was adjusted to 10ml/kg bodyweight and respiratory rate set to maintain end-tidal CO2 in the range 4.5–5.5%.

### *P*. *aeruginosa* Culture and Bead Preparation

*P*. *aeruginosa* embedded beads were prepared according to previously published methodology [16]. Briefly, broth cultures of *P*. *aeruginosa* mucoid strain PA0579 were prepared and cell suspensions mixed with molten agar before being injected into rapidly stirred heavy mineral oil. Beads were recovered thereafter by centrifugation.

### Bronchial Brush Biopsy

A bronchoscope (FG-16X; Pentax, Englewood, CO, USA) was advanced down the trachea and mainstem bronchi and then into the relevant segmental bronchi until the predefined area selected for brushing was identified. The protected specimen brush (ConMed Endoscopic Technologies, Disposable Microbiology Brush 130) was then advanced through the biopsy channel of the bronchoscope. The plug was expelled and the sheath was retracted before the brush was applied to the epithelial mucosal surface. By advancing and retracting the brush in contact with the mucosa, a sample of epithelial lining cells and fluid was obtained. The bronchial brush biopsy (BBr) sample was taken to a sterile flow cabinet and the brush end cut off directly into a non-stick Rnase free 1.5ml microfuge tube containing 1ml sterile phosphate buffered saline (Sigma D8537) and stored on ice. BBr samples were vortexed then the brush was removed under sterile conditions. Samples were centrifuged at 13000g for 15 minutes at 4°C and the pellet stored at -80°C.

### Bronchoalveolar Lavage

The bronchoscope was wedged in selected segmental bronchi. Two 20ml aliquots of PBS (Sigma D8537) were used to collect BALF from selected lung segments. BALF samples were placed into sterile Falcon tubes and immediately placed on ice until subsequent analysis. BALF was centrifuged at 400g for seven minutes to separate out the cellular fraction and the resultant pellet was re-suspended in 2ml sterile PBS. The total cell number was counted before subsequent preparation of cytospins for differential cytology. Cells were counted using a Neubauer haemocytometer and values expressed per millilitre BALF. Cyto-centrifuge slides were prepared and stained using Leishman stain for differential counts on 500 cells. Cells were classified as neutrophils, macrophages, eosinophils, lymphocytes or mast cells according to standard morphological criteria. The remaining BALF was centrifuged at 13000g for 5 minutes at 4°C and the pellet and supernatant stored at -80°C.

### Necropsy

Following euthanasia by intravenous injection of baribiturate, the heart and lungs were carefully removed from the carcase following standard necropsy protocols before the heart was dissected away and the lungs presented for further sampling and analysis. The trachea and right and left major bronchi were carefully opened along their dorsal aspect to expose the primary bronchial entrance to each lung segment of interest. Sterile polyethylene tubes were inserted in turn, into each lung segment bronchus until a wedge point was achieved. Thereafter 40ml sterile PBS (Sigma D8537) was instilled recovered and handled following the same principles employed during bronchoalveolar lavage under anaesthesia. Control lung segments were always sampled prior to handling segments previously exposed to Pseudomonas and all efforts were directed towards minimising the potential for cross-contamination between lung segments. Lung segments were carefully isolated by gross dissection from surrounding lung tissue before being separately examined and further dissected by parallel transverse sectioning along the plane of the subsegmental bronchus into ~1cm thick lung slices. Photographic images of the lung slices were collected. Samples were collected for assessing the degree of Pseudomonas infection.

### Pathology Grading

Photographic images of lung slices derived from each lung segment were assessed and scored for the presence (1) or absence (0) of the following gross pathological features–pleural oedema, pleural fibrosis, and the presence of fibrotic/granulomatous tissue or abscessation in the lung parenchyma. The cumulative score (range 0–4) for each slice image was then multiplied by the proportion, quantified using ImageJ ([18]), of the slice cut surface considered visibly abnormal to give a total pathology score for each segment. Scores were then normalised to a scale of zero (no pathology) to 100 (the most severe pathology) by dividing by the maximum score observed amongst all lung slices and multiplying by 100.

### Pseudomonas Infection Level

Tissue samples were stored on ice and then finely chopped under sterile conditions. 300mg of tissue was weighed and placed into Lysing Matrix D tubes (MP Biomedicals 6913–500) containing 600μl sterile PBS. Samples were homogenised using a Fastprep FP120 (Thermo Electron) with 3 bursts of 20 seconds at setting 6.0 and 5 minute incubation on ice between each homogenisation step. Equal volumes of BALF samples were centrifuged for 10 minutes at 2700g and each pellet re-suspended in 1ml sterile ice-cold PBS. BALF pellets were homogenised as for the tissue samples. Bacterial load was assessed from all samples by preparation of 10 fold dilutions in ice-cold PBS and 100μl of chosen dilutions spread on Pseudomonas Isolation Agar (PIA)(Sigma) plates and incubated overnight at 37°C. Bacterial counts were calculated by manual counting of colony forming units, multiplication by the dilution factor, and a further 10 fold, to allow for 100μl inoculum to give a final count in cfu/ml.

### Sensitivity

We assessed the sensitivity to CMS of the *P*. *aeruginosa* mucoid strain PA0579 used to infect the sheep, and isolates cultured from the infected lung tissue, using Etest strips (Etest, bioMérieux, France).

### DNA Isolation

DNA was extracted by modification of a previously published method [19]. Briefly, BBr pellets were suspended in 60μls of solution C1, provided with the PowerSoil DNA Kit (PowerSoil DNA Isolation Kit, MO-BIO). This suspension was transferred into PowerSoil Bead Tubes along with 750μl of PowerSoil Bead Solution. Bead Tubes were heated at 65°C for 10 minutes to aid cell lysis then placed in a FastPrep FP120 Cell Disrupter for 45 seconds at 5.0m/sec. All further steps were carried out following the manufacturer’s instructions except that the final elution volume was changed to 50μl rather than 100μl. Extracted DNA was stored at -80°C until used in a nested PCR with primers to amplify the hypervariable region V1-V4 of the 16S rRNA gene followed by bar coded primers to amplify the hypervariable V2-V3. The PCR products were Agencourt AMPure XP (Beckman Coulter) cleaned after each PCR run to remove smaller non-specific products and unused primers then the purified amplicon products from each sample were pyrosequenced.

Triplicate 20μl reactions were performed per sample using the LightCycler® 480 SYBR Green I Master mix (Roche), 1μl of extracted DNA solution and the 16S rRNA Q-PCR primers UniF340 (5’–ACTCCTACGGGAGGCAGCAGT–3’) and UniR514 (5’–ATTACCGCGGCTGCTGGC–3’) at a final concentration of 0.4μM. The following steps were performed: a pre-incubation step of 50°C (ramp rate: 4.80°C/s for 2 minutes) then 95°C (ramp rate: 4.80°C/ for 10 seconds) and an amplification step consisting of 45 cycles of 95°C (ramp rate: 4.80°C/s for 30 seconds) then 63°C (ramp rate: 2.50°C/s for 30 seconds). This was followed by a melting cycle consisting of 95°C (ramp rate: 4.80°C/s for 5 seconds) then 65°C (ramp rate: 4.80°C/s for 1 minute) followed by 97°C (ramp rate: 0.11°C/s, acquisition mode, continuous). A standard curve beginning at 1.7 × 10^9^ copies in nuclease free water and continuing in 1:10 dilutions to 1.7 × 100 was generated from a PCR product obtained from the genomic DNA of *P*. *aeruginosa* mucoid strain PA0579 [20] using the 16S rRNA primers 28F and 805R (PCR method described below), in order to calculate the total 16S rRNA gene copy numbers.

### Barcoding of 16S Amplicons and Pyrosequencing

All PCR steps used 25μls of PCR master mix (Q5® High-Fidelity 2X Master Mix, New England Biolabs) and 2.5μls of both forward and reverse primers. A nested protocol of two rounds of PCR reactions was performed. A negative control was included in each PCR run consisting of nuclease free water.

The V1-V4 variable region of the bacterial 16S rDNA was amplified using the primers 28F (5’–GAGTTTGATCNTGGCTCAG–3’) and 805R (5’–GACTACCAGGGTATCTAATC–3’). The conditions for the first round of PCR were: 94°C for 2 minutes followed by 30 cycles of 94°C for 1 minute, 55°C for 45 seconds and 72°C for 1.5 minutes followed by 72°C for 20 minutes.

The V2-V3 region was amplified using Truseq barcoded primers 104F (5’—GGACGGGTGAGTAACACGTG–3’) and 519R (5’–GTNTTACNGCGGCKGCTG–3’)[21]. The conditions for the second round of PCR were: 98°C for 30 seconds followed by 20 cycles of 98°C for 10 seconds, 67°C for 30 seconds and 72°C for 10 seconds followed by 72°C for 2 minutes. Amplicons from both rounds of PCR were purified using the AMPure XP system (AMPure XP PCR Purification, Agencourt).

Amplicons were sequenced using an Illumina MiSeq producing paired 250-nucleotide reads [22]. Two negative PCR controls were included in the sequencing run.

### Data Analysis

Primers were removed using Cutadapt [23]. The MOTHUR program [24] was used for quality control and taxonomic assignment of reads, following a protocol developed for MiSeq by the MOTHUR creators [22]. Sequences were phylotyped using the SILVA reference alignment and any sequences which did not correctly align were removed. Chimeras were identified and removed using UCHIME [25] within MOTHUR. Sequences were taxonomically classified, using MOTHUR’s Bayesian classifier, against the Greengenes database [26]. Quality control consisted of the removal of sequences if they were not assigned to bacteria; were identified as chimeric; contained ambiguous bases or homopolymers < 9 bases; did not align to the correct region of the 16S gene or were less than 359 bases long. All samples were found to have Good’s coverage values greater than 0.99, indicating sequencing to sufficient depth for the purposes of this study.

For quality control purposes, water samples were sequenced and analyzed through the bioinformatics pipeline. These samples had a much less diverse microbial community composition with over 66% abundance accounted for by only three OTUs. Whilst these OTUs could also be found in lung microbiota samples their proportional abundance was typically much less with median [range] proportional abundances of 0.4 [0–6.5], 0.0 [0–14.5], and 0.0 [0–3.4] respectively. No specific adjustment in analysis was made for OTUs present in water control samples. Sequence data was submitted to the Sequence Read Archive (http://www.ncbi.nlm.nih.gov/sra; accession number SRP064022).

### *P*. *aeruginosa* Specific qPCR

Targeting the oprl gene was performed following a modification of a previously published method [27]. Briefly, 1μl DNA samples were run in triplicate with 12.5μl Qiagen Quantitect probe mastermix, 0.3μM of each OPRL primer and 0.2μM hydrolysis probe with the reaction made up to a final volume of 25μl with water. Gene copy number was calculated from a standard curve of genomic DNA of *P*. *aeruginosa* mucoid strain PA0579.

### Statistical Approach

Where data was normally distributed parametric data analysis procedures were applied (t-test, ANOVA), otherwise non parametric alternatives (Mann Whitney Test, Kruskal Wallis Test, Wilcoxon Signed-Ranks test and Spearman rank correlation) were used. Heatmaps were generated using the “heatmap.2” R package, version 2.10.1 (available at http://CRAN.R-project.org/package=gplots). Non-metric multidimensional scaling (NMDS) analysis applied to distance matrices of Bray-Curtis dissimilarities, and ordination plots were generated using the “vegan” R package. Permutational ANOVA (PERMANOVA) (using the R-vegan function adonis) on Bray-Curtis distance matrices facilitated analysis and partitioning of sums of squares.

## Results

### Clinical Response

There were no signs of adverse effect nor significant variation in bodyweight in any sheep during the experimental period. All rectal temperatures lay within the range 39.0–40.2 (normal range 37.9–40.3, n = 664; D Collie, unpublished observations) and there was no significant difference between the groups.

### Routine Haematology

Instillation of P aer. was associated with a significant reduction in the total white blood cell count in peripheral blood measured three days after the first instillation, a reduction that reverted to baseline levels by fourteen days after the first instillation (ANOVA; p = 0.000, relative to baseline and to day 14). There was no difference between the groups in this respect. There was no significant change in any other measured haematological parameters.

### Bronchoalveolar Lavage Cytology

Infection with P aer. was associated with an increase in bronchoalveolar cellularity. The predominant cell types involved comprised alveolar macrophages, neutrophils and lymphocytes. Bubble plots illustrating the relationship between the log-transformed absolute numbers of these cell types (in baseline and post-infection samples from directly infected and remote lung segments) and the level of infectivity in the same samples are depicted in S1 Fig. Cytospin images representative of baseline and post-infection cytology are also depicted in S2 Fig.

### Bacterial Load in BAL and Tissue Specimens

**Fig 2:**
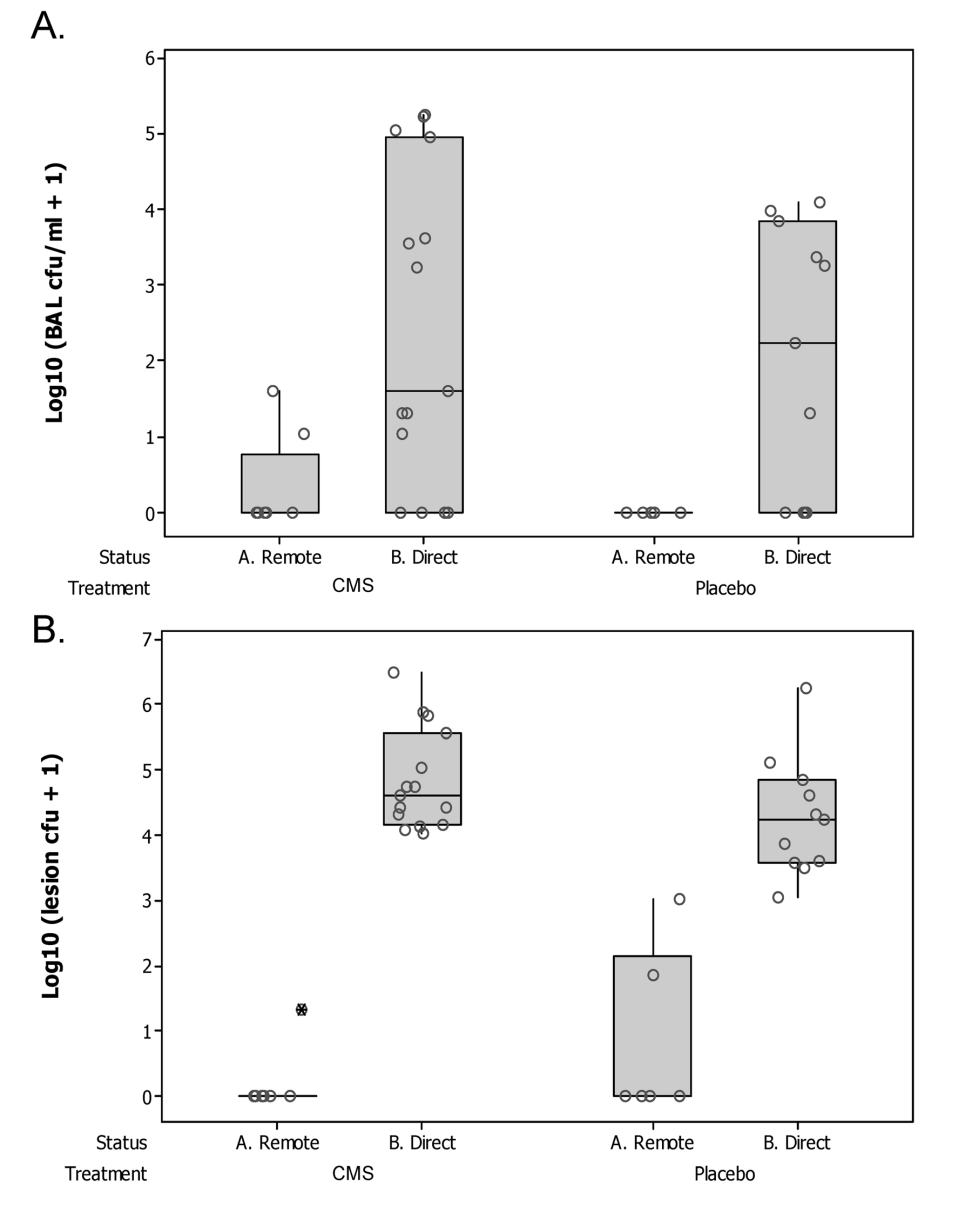
Bacterial burden in the lung. Boxplots showing the bacterial burden in A. bronchoalveolar lavage fluid (Log_10_(BAL cfu/ml + 1) and B. lung tissue (Log_10_(lesion cfu+1)) samples derived from lung segments directly infected 14 days previously with 2.5x10^9^ cfu *P*. *aeruginosa* in agar beads (B. Direct), and lung segments spatially remote to such segments (A. Remote). Sheep were treated with daily intravenous CMS (n = 4), or placebo (n = 4), between days 7–14. Bronchoalveolar lavage fluid samples derived from direct lung segments had a significantly greater burden of *P*. *aeruginosa* than remote lung segments (Wilcoxon Signed Rank Test of median = 0 versus median > 0; P = 0.030)); there was no significant difference between the placebo and CMS groups in this regard. Lung tissue samples derived from direct lung segments had a significantly greater burden of *P*. *aeruginosa* than remote lung segments (Wilcoxon Signed Rank Test of median = 0 versus median > 0; P = 0.007); there was no significant difference between the placebo and CMS groups in this regard.

P aer. infection was reliably detected in all direct lung segments by culture of BALF and/or lung tissue. Whilst BALF samples derived from direct lung segments had a significantly greater burden of P aer. than remote lung segments (Wilcoxon Signed Rank Test of median = 0 versus median > 0; P = 0.030))(Fig 2A) there was no significant difference between the placebo and CMS groups in this regard. Two BALF samples derived from remote lung segments (2S065_RA and 2S037_RVD1 (CMS)) demonstrated evidence of infection. We also examined tissue from lung segments for evidence of infection and again found that samples derived from direct lung segments had a significantly greater burden of P aer. than remote lung segments (Wilcoxon Signed Rank Test of median = 0 versus median > 0; P = 0.007)(Fig 2B) with no difference between the groups. Three tissue samples derived from remote lung segments (2S037_RVD1 (CMS) and 2D616_RA & RVD1 (Placebo)) demonstrated evidence of infection. The *P*. *aeruginosa* mucoid strain PA0579 used to infect the lung was sensitive to CMS, and we found no difference between the infecting strain, and isolates cultured from lung tissue obtained at necropsy, in this respect (data not shown). In addition colony morphotypes were, with one exception, uniform in appearance and mucoid in character. The exception concerned colonies grown from tissue samples derived from a directly infected lung segment (LVD2) of a sheep treated with CMS (2D615). In this instance there was evidence of non-mucoid colonies intermixed with mucoid colonies.

### Pathology

Semi-quantitative scoring of the gross pathological features indicated that there was a significant increase in gross pathology associated with direct infection with P aer. (Wilcoxon Signed Rank Test of median = 0 versus median > 0; P = 0.007) and that there was no difference between placebo- and CMS-treated sheep in this respect. Notably, with only one exception all the remote segments scored zero (the exception scoring 1), whereas the median [range] for the placebo sheep was 60.5 [12.3–95.0] and for the CMS sheep 30.5 [19.3–100.0]. There was a significant correlation between the pathology score and the burden of infection in BALF and tissue (Spearman Rho = 0.625 & 0.731, and P = 0.017 & 0.001 respectively).

### Microbiota

**Fig 3:**
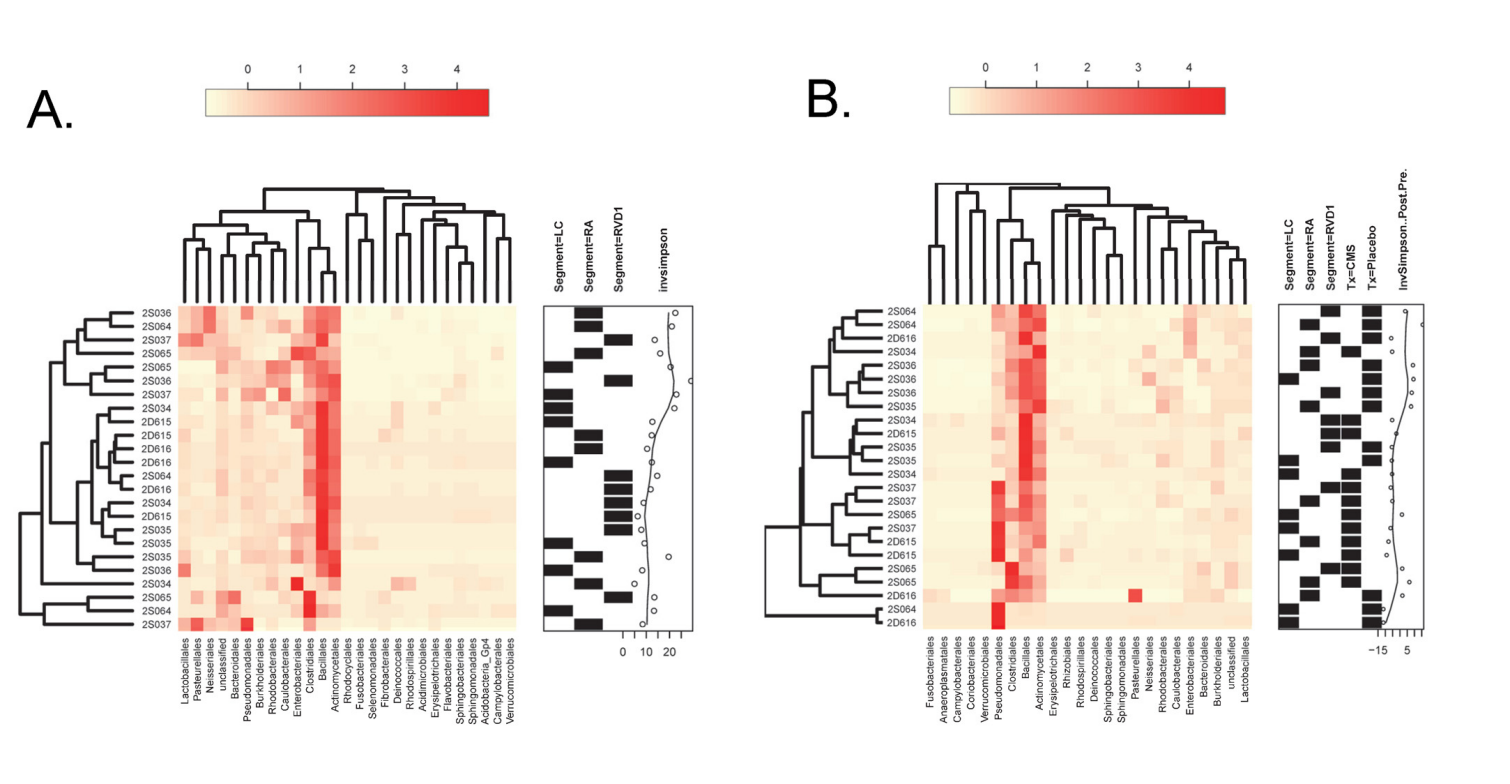
Abundance heatmaps for Pre- and Post-data. Heatmaps reflecting the proportional representation of microbiota (Order classification) in samples derived from different lung segments at baseline (A), and after lung infection and/or treatment with systemic CMS (B). The identities of individual sheep are indicated at the left side of each heatmap and the segment from which each sample was derived (LC, RA or RVD1) indicated in the annotation frame on the right side of the heatmap. Orders with a proportional representation of less than 1% are not shown. The results of hierarchical clustering applied to a distance matrix of Bray-Curtis measures between pairs of samples is shown for samples (left side) and bacteria (top). The remaining annotation in (A) reflects the inverse Simpson index that characterizes the species diversity in each sample community, with higher values reflecting an increase in diversity. In (B) the annotation frame also indicates whether the sample was derived from a sheep treated with CMS (Tx = CMS) or placebo (Tx = Placebo), and the change in the inverse Simpson index (Post-Pre) relative to the values at baseline, with positive values reflecting an increase, and negative values a decrease, in diversity.

The heatmap in Fig 3A illustrates that in the baseline (Pre) samples the most predominant consistently represented taxa were Bacillales (25+14% [1–49])(Mean+SD [Range]), Actinomycetales (18+9% [1–37]) and Clostridiales (14+10% [2–45]). Other taxa such as Enterobacteriales, Bacteroidales, Caulobacterales, Pasteurellales and Pseudomonadales also featured prominently but inconsistently.

**Fig 4:**
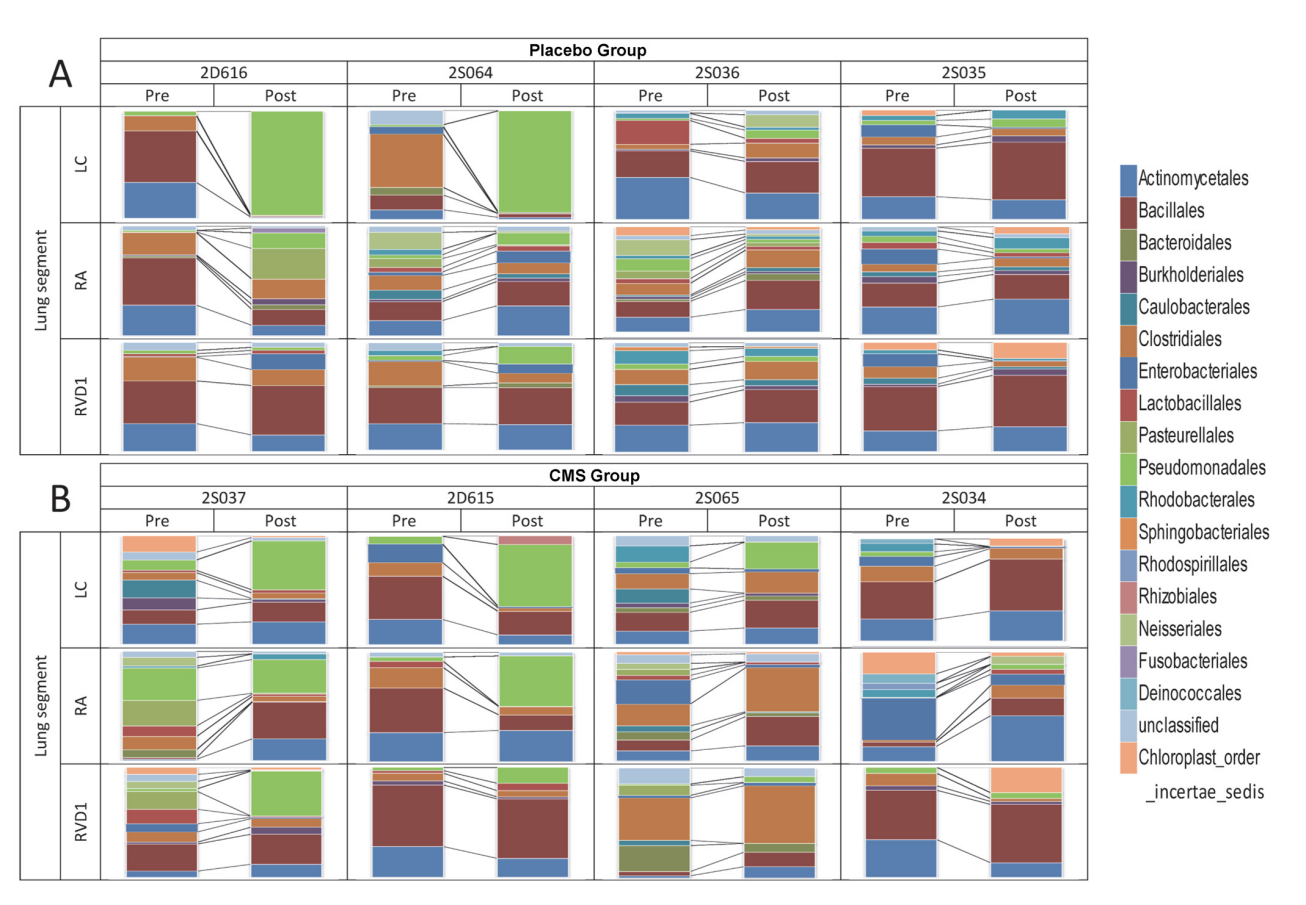
Proportional changes in microbiota from paired Pre- and Post- samples. Stacked column charts depicting the relative proportions of different bacterial phylotypes (classified at the level of Order, and coloured according to the legend) in PSB samples derived from three lung segments (Left cardiac (LC), Right apical (RA) and Right ventral diaphragmatic (RVD1)) prior to (Pre) and 14 days after (Post) the initiation of chronic lung infection with *P*. *aeruginosa* in segment LC. Four sheep (2D616, 2S064, 2S036 and 2S035) were treated with daily intravenous injections of saline (A. Placebo Group), and four sheep (2S037, 2D615, 2S065 and 2S034) were treated with daily intravenous injections of CMS (B. CMS Group) between days 7–14.

**Fig 5:**
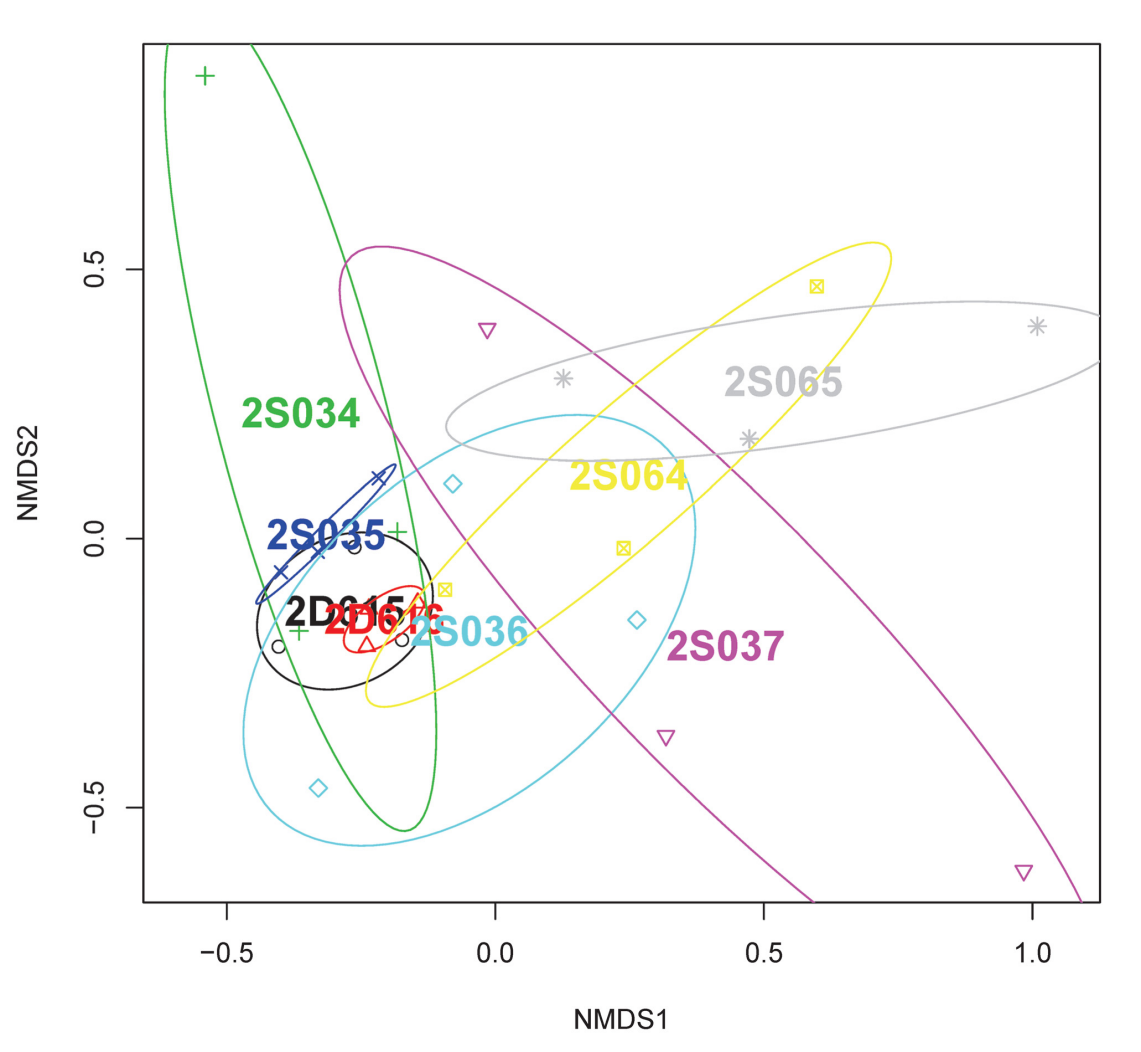
Non-metric multi-dimensional scaling (NMDS) ordination of bacterial communities in the lungs of healthy sheep. Individual plot data is grouped according to sheep identity and can be related through shared colour, a process facilitated by the coloured ellipses. Whilst some sheep have microbial communities that cluster tightly, and others are widely scattered, there is considerable overlap apparent between most of the sheep.

For three sheep (2D616, 2S035 and 2D615) the predominant pattern was consistently represented in the samples derived from three different lung segments (Fig 4A), demonstrating within-lung homogeneity of microbiota. The remaining samples were found in sheep in which microbial communities were more diverse, demonstrating within-lung heterogeneity of microbiota. Visualisation of non-metric multi-dimensional scaling (NMDS) ordination analysis applied to the distance matrix of Bray-Curtis dissimilarities confirmed the above visual perceptions (Fig 5).

We performed hierarchical cluster analysis to ascertain whether samples would cluster by sheep and/or lung segment (Fig 3A) and employed permutational ANOVA (PERMANOVA) using the Bray-Curtis distance matrix to assess the extent to which variability could be assigned to the sheep, or lung segment, from which the communities were drawn. The results of the latter analysis indicated that a significant proportion of the variance (49%) could be explained by a sheep effect (P = 0.003), whereas the lung segment had no significant effect (P = 0.155).

The inverse Simpson index was calculated for each of the samples (Fig 3A). This index takes into account both species richness, and evenness of abundance among the species present. In essence it measures the probability that two entities taken at random from the dataset of interest represent the same type. Values ranged from 5.0 to 29.5, with an average of 14.1 and SD 6.25. There was no significant relationship between particular lung segments and the diversity of microbiota contained therein as measured by the Simpson Diversity Index (Kruskal Wallis Test, P = 0.735).

All sheep (n = 8) were then subjected to chronic local lung infection with *P*. *aeruginosa*. Four sheep were systemically treated with intravenous CMS and four treated with a placebo injection. The heatmap in Fig 3B illustrates that the four most predominant orders in the Post- samples consisted of Bacillales, Actinomycetales, Clostridiales and Pseudomonadales.

Fig 4B depicts the proportional representation of different phylotypes in post-infection (Post) samples derived from direct (LC) and remote (RA & RVD1) lung segments of each sheep and the relationship of these samples to their baseline (Pre) counterparts.

Hierarchical cluster analysis of the Post- samples failed to indicate clustering according to sheep, lung segment, treatment (placebo or CMS), or any change in diversity as reflected in the Post-Pre inverse Simpson Diversity index (Fig 3B). PERMANOVA analysis indicated that a significant proportion of the variance (42%) for Post- samples could be explained by a sheep effect (P = 0.01), and whether or not the segment had been directly infected (12%; P = 0.01). Whether or not the sheep had been treated with CMS or placebo had no significant bearing on the variance in the Post- samples.

**Fig 6:**
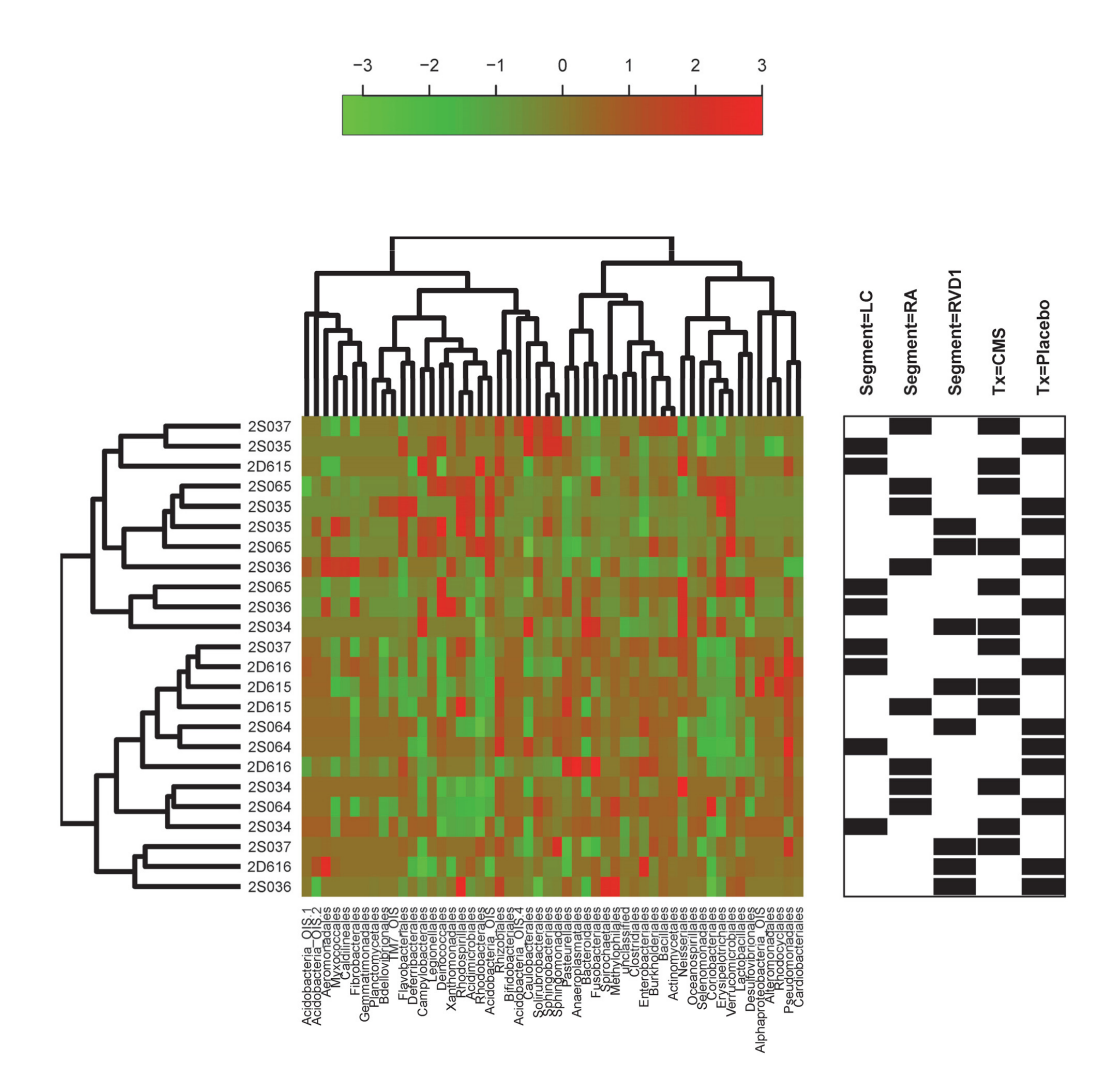
Fold change heatmap. Heat map representing the Log_2_(Post-/Pre-) fold-change for the phylotypes in each set of paired samples. The degree of relatedness between different samples was assessed by creating a distance matrix of Pearson correlation coefficients using the formula (1-Pearson Correlation Coefficient) as the index of dissimilarity. Hierarchical clustering failed to indicate any overarching influence of treatment, or lung segment, on the elicited patterns of change.

We calculated the Log_2_(Post-/Pre-) fold-change for the phylotypes in each set of paired samples (Fig 6) and examined the degree of relatedness between different samples by creating a distance matrix of Pearson correlation coefficients using the formula (1-Pearson Correlation Coefficient) as the index of dissimilarity. Hierarchical clustering failed to indicate any overarching influence of treatment, or lung segment, on the elicited patterns of change. PERMANOVA applied to a Euclidean distance matrix based on the sample-specific arrays of fold-changes failed to highlight any significant contributor to the variance observed.

**Fig 7:**
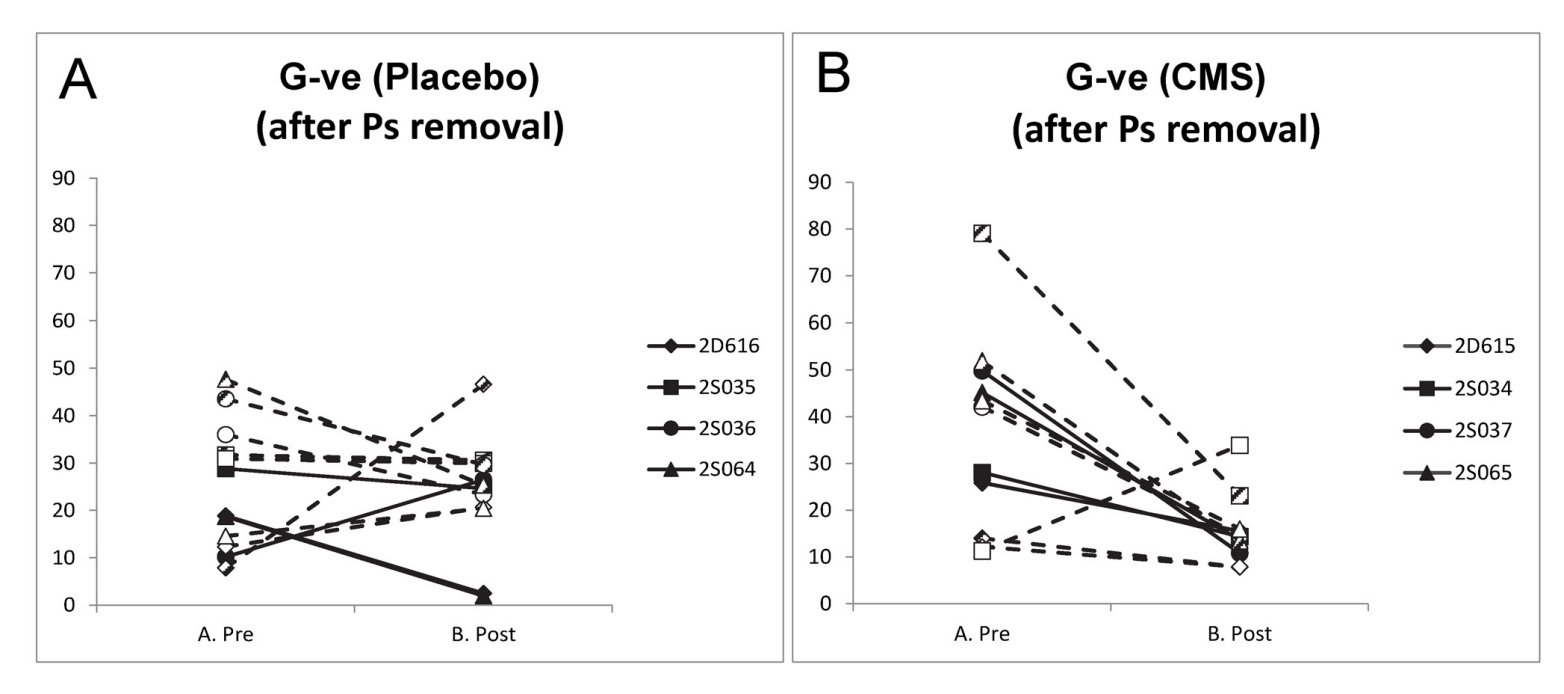
Change in Gram negative bacteria (excluding Pseudomonadales) in Pre- and Post-samples. Line charts indicating the percentage of Gram negative bacteria in PSB samples derived prior to (Pre) and 14 days after infection with P.aer (Post) from sheep treated with daily intravenous injections of A. saline (Placebo), or B. CMS (CMS) between days 7–14. Symbols reflect the identity of sheep according to the legend entries, with filled symbols reflecting samples derived from directly infected lung segments (LC), partially filled symbols from remote segment RA, and open symbols from remote segment RVD1.

We determined whether systemic therapy with CMS had any influence on the proportion of Gram negative bacteria (excluding Pseudomonadales) in lung microbiota. Whereas the presence of local lung infection with Ps aer did not significantly alter the proportion of Gram negative bacteria (excluding Pseudomonadales)(Fig 7A) in the lung segments of sheep treated with placebo (Paired t-test on Pre-Post differences (n = 4), P = 0.741), all of the direct segments and all except one of the remote segments (7/8) of sheep treated with CMS experienced a reduction in the proportion of Gram negative bacteria (excluding Pseudomonadales)(Fig 7B). The reduction in the proportion of G-ve bacteria (excluding Pseudomonadales) in lung segments of the CMS group was significant (Paired t-test on Pre-Post differences (n = 4), P = 0.040). When only the remote lung segments were included in this analysis, again the proportion of Gram negative bacteria (excluding Pseudomonadales) showed no significant change in response to infection in the placebo group whereas there was a significant reduction in this proportion in the sheep treated with CMS (Paired t-test on Pre-Post differences (n = 4), P = 0.979, and P = 0.044 respectively).

We determined, for the same samples, whether there was a relationship between qPCR for PA0579 and reads assigned to Pseudomonadales following 16SrRNA sequencing. There was a highly significant positive correlation (Spearmans rho = 0.711 (n = 19, p<0.001)).

## Discussion

Instillation of *P*. *aeruginosa* in agar beads consistently induced a chronic local lung infection that resulted in gross pathology that remained confined to the areas where the instillate was delivered. The gross pathological features and changes in bronchoalveolar cytology were consistent with our previous experience with this model system [16].

We were able to assess the composition of lung microbiota across the lung by sampling from different airways of the same animals at the same point in time and to compare these samples amongst different animals.

We found that the microbial communities of the samples obtained at baseline were largely dominated by the orders Bacillales, Actinomycetales and Clostridiales. Whilst there was evidence for both within-lung homogeneity and heterogeneity amongst different animals, samples obtained from a given sheep were more likely to share similarity with other samples from the same sheep than from samples from different sheep.

Lung microbiota data derived from healthy human subjects is becoming increasingly available [19, 28, 29]. Charlson et al (2011) found that the predominant orders represented in BALF and PSB samples were Bacteroidales (dominated by Prevotella spp.), Clostridiales, Lactobacillales (dominated by Streptococcal spp.) and Actinomycetales [19]. Hilty et al (2010) [30] similarly found that samples from healthy human subjects (adults and children) were dominated by Bacteroidales (Prevotella spp.), as did Dickson et al (2015) [29]. Indeed it is perhaps the prevalence of Bacteroidales, and consistent presence of Lactobacillales, that links these different studies of healthy human subjects. Otherwise, these separate studies are notable for their respective differences that are largely unexplained–such as the notable presence of Methylobacterium (Order: Rhizobiales) in the recent study of Dickson et al (2015) [29].

Bacteria of the order Baccillales, the most predominant members of the sheep lung microbiota, are therefore relatively infrequently found in human lungs whilst bacteria belonging to the other predominant orders, Actinomycetales and Clostridiales, can be found in human lungs but are inconsistently present.

The relatively minor contribution of Bacteroidales to ovine lung microbiota (~5%) is an interesting observation given both the predominance of Prevotella in the human lung and its high abundance in the rumen of sheep [31] where they help the breakdown of protein and carbohydrate foods. If measured lung microbiota in sheep are derived transiently from the oropharynx (itself heavily influenced by rumen contents through the process of rumination) then it would be reasonable to assume that Bacteriodales would feature more prominently.

Notably Dickson et al (2015) [29], in exploring whether the lung microbiome is spatially varied in healthy adults, determined that intrapulmonary sites, when compared to each other, did not contain consistently distinct microbiota, but that intra-subject variation was significantly less than inter-subject variation. We similarly established that whilst sheep lung segment had no significant bearing on the composition of microbiota, sheep identity did have a significant impact. Coupling the latter finding with the sometimes observed high degree of within-lung heterogeneity is conceptually difficult and raises obvious questions regarding both the spatial extent of distinct microbial communities and their longitudinal stability. Indeed, whilst we sampled from disparate lung segments and found heterogeneity in some sheep it is conceivable that in these animals PSB samples from neighbouring bronchi within the same segment, or even different locations along the same bronchi, might also reflect heterogeneity. Equally we currently have no idea whether lung microbiota ‘states’ are constant within individual sampling sites over time. Addressing such questions will be fundamental to developing a hypothetical modelling framework for ovine lung microbiota that captures both individual identity and potential within-individual heterogeneity at a given point in time.

Dickson, Erb-Downward and Huffnagle (2014)[32] highlighted in their recent review that the invasive nature of microbiota sampling in the lower respiratory tract has hitherto precluded the gathering of data to assess the extent of temporal and spatial heterogeneity of the lung microbiome in healthy human subjects. We would contend that large animal models offer the facility to probe such relationships and develop experience and methodology that will potentially impact on our ability to understand the relevance of change in the composition of lung microbiota in humans.

Whilst much interest surrounds the relationship between lung microbiota states and diseases such as asthma, COPD and CF, these studies, by their nature, only reflect associations. If such observations are to be usefully extended and the functional significance of lung microbiota established then there is a clear need to develop animal models of lung microbiota states to test mechanistic hypotheses [32].

This model system provided us with the opportunity to determine, in the first instance, whether local lung infection with *P*. *aeruginosa* would alter lung microbiota, as reflected in PSB samples, in both the direct lung segments and in areas of the lung ‘remote’ to those segments. Whilst lung infection resulted in consistent lung pathology PSB samples from only two of the direct segments in the placebo group demonstrated a heavy proportional burden of Pseudomonadales. It is worth noting that the airways from which these samples were derived could not be visually differentiated from airways yielding lesser burdens at bronchoscopy (D Collie personal communication). The highly significant positive correlation between qPCR for PA0579 and reads assigned to Pseudomonadales following 16SrRNA sequencing indicates the likelihood that the heavy proportional burden of Pseudomonadales represented the infecting strain, PA0579.

Whilst the failure to demonstrate Pseudomonadalesin PSB samples derived from some subsegmental bronchi serving lung segments with obvious gross pathology and chronic *P*. *aeruginosa* lung infection presumably reflects the particular pathophysiology underlying these instances, the lack of relationship highlights important caveats in interpreting microbiota changes in our model of lung infection–that PSB microbiota relate only to the precise location wherefrom the sample was derived, and that lung pathology and infection may be highly locally compartmentalised and closely juxtapose airways with minimal evidence of infection.

We also established that local lung infection was not associated with any uniform ‘lung-wide’ change in lung microbiota sampled from areas distant to that infection. However it was apparent that an increase in the proportion of Pseudomonadales occurred in three of the four remote segments of the two sheep that demonstrated a profound increase in the proportion of Pseudomonadales in their direct lung segments, whereas the sheep that failed to demonstrate an increase in proportion of Pseudomonadales in their direct lung segments also failed to show any appreciable change in the proportion of Pseudomonadales in any of their remote lung segments.

We demonstrate in this ovine model of chronic local lung infection with *P*. *aeruginosa* that 7 days of once-daily intravenous treatment with CMS had no effect on the burden of *P*. *aeruginosa* infection in the directly infected lung segments. It is considered that both the nature of the lung pathology and the pharmacodynamics of CMS in sheep following intravenous delivery will have conspired to undermine any therapeutic effect of CMS.

The model protocol, in seeding *P*. *aeruginosa* in agar beads and delivering these beads to lodge in the distal airways and lung parenchyma evoked a vigorous local inflammatory and immune response that could not be fully resolved. The protective agar matrix, together with abscessation and fibrosis, in representing the body’s attempts to limit spread of infection from these chronic nidi of infection, would also potentially hinder access of therapeutics.

CMS has a concentration-dependent effect on Gram negative bacteria. To be effective it must achieve bactericidal peak tissue concentrations in infected lung parenchyma and/or the airway epithelial lining fluid. Whilst we measured neither in this study it is considered unlikely that the dose, mode and frequency of therapy would have generated bactericidal concentrations of CMS in the epithelial lining fluid of the airways. At the inception of this study there was, to the authors’ knowledge, no available data concerning the pharmacokinetics of CMS following intravenous administration in sheep, and neither was any specific published guidance available concerning potential toxicity in this species following intravenous delivery. Our choice of dose, mode and frequency of therapy were therefore driven by pragmatic considerations. More recent data does however confirm that CMS is indeed not detected in airway epithelial lining fluid after intravenous dosing in sheep [33]. Further, whilst Boisson et al (2014)[34] did demonstrate an increase in ELF concentration of CMS and colistin after IV administration in critically ill patients, Imberti et al. (2010)[35], following intravenous administration of CMS to adult patients with ventilator-associated pneumonia caused by Gram-negative bacteria, could not measure CMS in BAL fluid. The results of both studies inferred a likely deficiency in therapeutic effect in the lung following intravenous delivery.

In line with these considerations, sheep that were treated with systemic CMS demonstrated a similar spectrum of microbiota change as was seen in the placebo group and there was no evidence of a specific effect of CMS on the proportion of Pseudomonadales detected in PSB samples. However, despite this apparent lack of effect and the unfavourable pharmacokinetic/pharmacodynamic characteristics of intravenous CMS in sheep, therapy did reduce the proportion of Gram negative bacteria (other than Pseudomonadales) and therefore increased the relative proportion of Pseudomonadales to other Gram negative bacteria in these samples. That sub-therapeutic doses can have such an effect may hold relevance when viewed in the context of potentially changing ecological niche characteristics across the whole lung to those more favourable to the survival of *P*. *aeruginosa*. Indeed, Rogers et al (2014) demonstrated that in non-CF bronchiectasis patients without *P*. *aeruginosa* airway infection, erythromycin did not significantly reduce exacerbations and promoted displacement of *Haemophilus influenzae* by more macrolide-tolerant pathogens including *P*. *aeruginosa* [14].

Despite, in some instances, evidence of considerable flux in lung microbiota between baseline and 14 days after establishing chronic local lung infection with *P*. *aeruginosa*, sheep could not be distinguished on the basis of observed clinical response. Whilst the inference might therefore be construed that local lung microbiota have no functional impact on the pathophysiology of PEs this would be presupposed on the validity of this system to reliably model the pathophysiology and clinical features of PEs in humans. Whilst this presumption has yet to be fully explored in this system, the need to develop novel model systems whereby the pathophysiology of PEs can be investigated means that such studies must remain an imperative.

In conclusion, sheep lung microbiota are dominated in health by bacteria belonging to the orders Bacillales, Actinomycetales and Clostridiales. Whilst chronic local lung infection with *P*. *aeruginosa* led to increased predominance of Pseudomonadales, such predominance was not uniformly consistent amongst either directly infected or remote lung segments. Treatment of sheep with daily intravenous CMS, whilst failing to overtly influence lung microbiota, did significantly increase the proportion of Pseudomonadales relative to other Gram negative bacteria in infected sheep.

## Supporting Information

S1 Fig Bacterial burden and bronchoalveolar lavage fluid cellularity. Bubble plots depicting the relationship between bronchoalveolar lavage fluid cellular composition and bacterial burden in samples derived at baseline (A. Pre) and after infection, both from areas of the lung subject to direct lung infection with P. aeruginosa (C. Post-direct) and areas remote to those sites (B. Post-remote). Bubbles are coloured according to the sheep to which they relate and the legend further specifies to which treatment group the sheep belong (Treatment_Sheep). The size of each bubble relates to the bacterial burden present in that particular sample and can be gauged through reference to the legend (BAL cfu/ml). Data relating to the log-transformed absolute number of A. alveolar macrophages (Log10 (ABS AMs+1), B. neutrophils (Log10 (ABS Neuts+1), C. lymphocytes Log10 (ABS Lymph+1), D. mast cells Log10 (ABS Mast+1) and E. eosinophils Log10 (ABS Eosin+1) are shown.
(TIFF)

S2 Fig Bronchoalveolar lavage fluid cytology. Cytospin images representative of baseline (A) and post-infection (B) cytology. Alveolar macrophages were the predominant cell type present in baseline samples. Following the establishment of lung infection the proportion of neutrophils (arrowheads) and lymphocytes (*) increased in the directly infected lung segments.
(TIFF)
