# Supplementary figures and images for: Lung Microbiota Changes Associated with Chronic Pseudomonas aeruginosa Lung Infection and the Impact of Intravenous Colistimethate Sodium
Source: PLoS One. 2015 Nov 6;10(11):e0142097. doi: 10.1371/journal.pone.0142097 (PMC4636361; doi:10.1371/journal.pone.0142097)

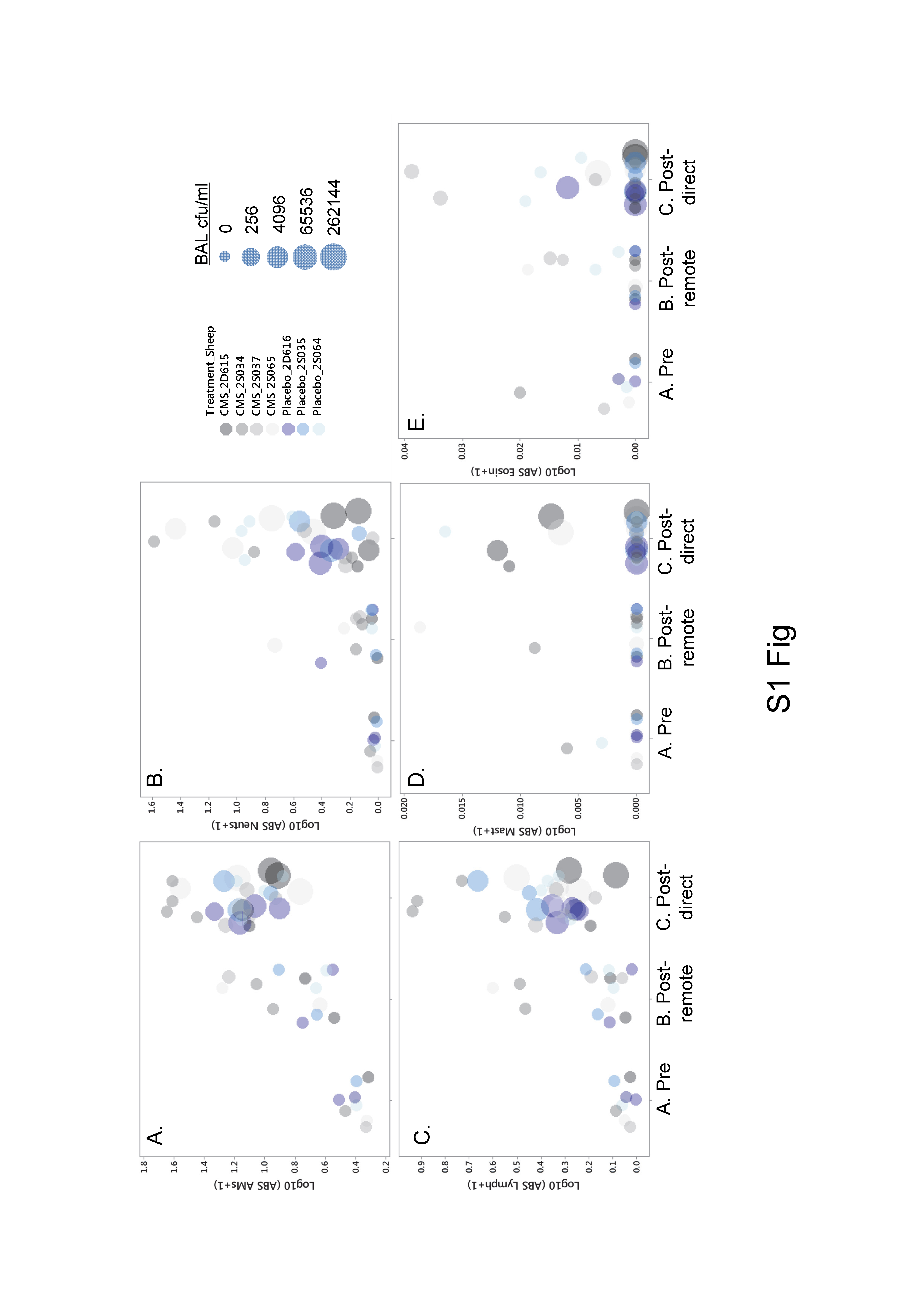

Supplement: S1 Fig — Bubble plots depicting the relationship between bronchoalveolar lavage fluid cellular composition and bacterial burden in samples derived at baseline (A. Pre) and after infection, both from areas of the lung subject to direct lung infection with P. aeruginosa (C. Post-direct) and areas remote to those sites (B. Post-remote). Bubbles are coloured according to the sheep to which they relate and the legend further specifies to which treatment group the sheep belong (Treatment_Sheep). The size of each bubble relates to the bacterial burden present in that particular sample and can be gauged through reference to the legend (BAL cfu/ml). Data relating to the log-transformed absolute number of A. alveolar macrophages (Log10 (ABS AMs+1), B. neutrophils (Log10 (ABS Neuts+1), C. lymphocytes Log10 (ABS Lymph+1), D. mast cells Log10 (ABS Mast+1) and E. eosinophils Log10 (ABS Eosin+1) are shown. (TIFF) [file pone.0142097.s001.tiff]

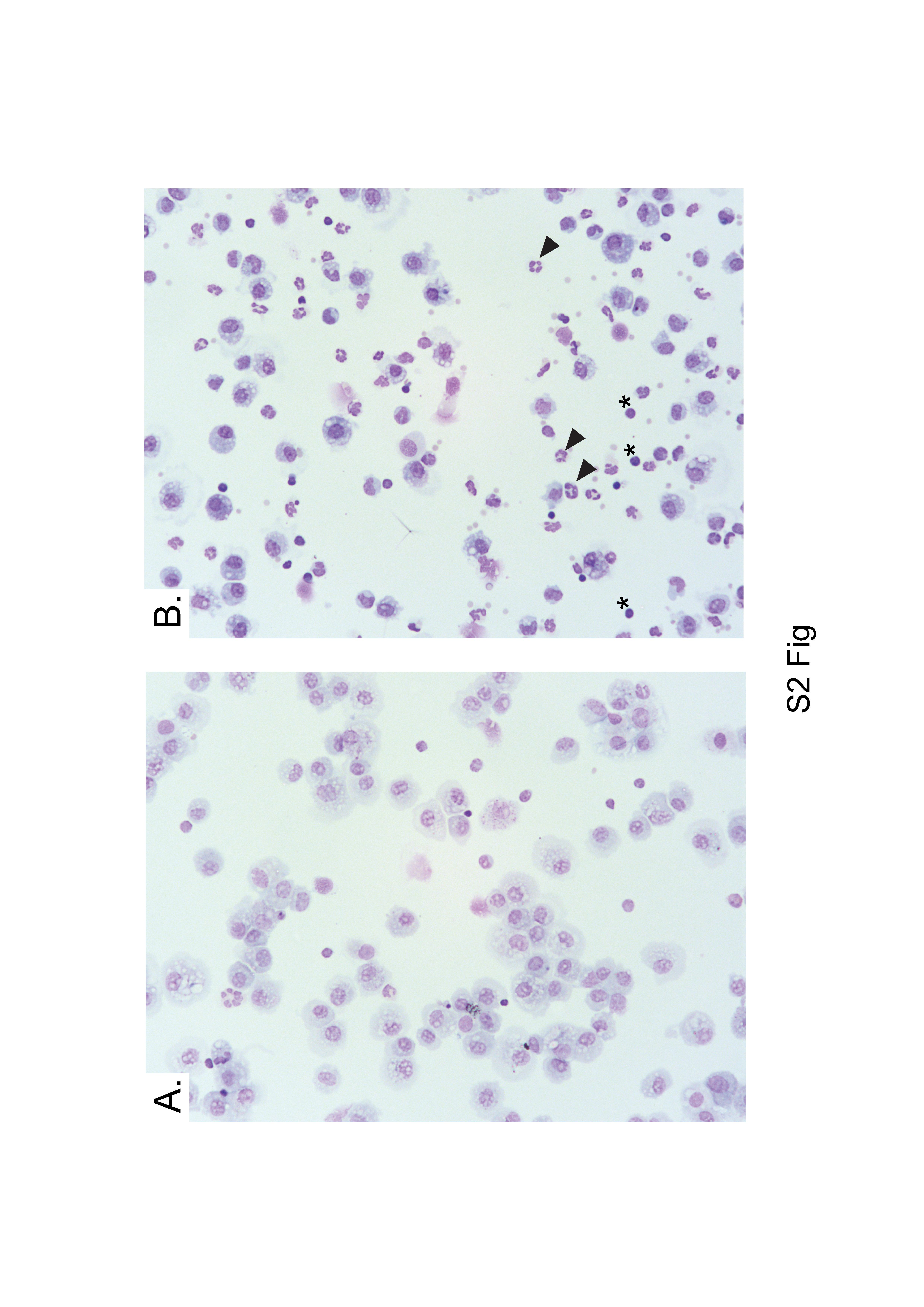

Supplement: S2 Fig — Cytospin images representative of baseline (A) and post-infection (B) cytology. Alveolar macrophages were the predominant cell type present in baseline samples. Following the establishment of lung infection the proportion of neutrophils (arrowheads) and lymphocytes (*) increased in the directly infected lung segments. (TIFF) [file pone.0142097.s002.tiff]
